# A Hybrid Model for 30-Day Syncope Prognosis Prediction in the Emergency Department

**DOI:** 10.3390/jpm14010004

**Published:** 2023-12-20

**Authors:** Franca Dipaola, Mauro Gatti, Roberto Menè, Dana Shiffer, Alessandro Giaj Levra, Monica Solbiati, Paolo Villa, Giorgio Costantino, Raffaello Furlan

**Affiliations:** 1 Internal Medicine, Syncope Unit, IRCCS Humanitas Research Hospital, 20089 Milan, Italy; raffaello.furlan@hunimed.eu; 2 IBM, 20100 Milan, Italy; mauro_gatti@it.ibm.com; 3 Department of Medicine and Surgery, University of Milano-Bicocca, 20100 Milan, Italy; r.mene@campus.unimib.it; 4 Emergency Department, IRCCS Humanitas Research Hospital, 20089 Milan, Italy; dana.shiffer@humanitas.it; 5 Department of Biomedical Sciences, Humanitas University, 20072 Milan, Italy; alessandro.giajlevra@humanitas.it; 6 Emergency Department, Fondazione IRCCS Ca’ Granda Ospedale Maggiore Policlinico, Università Degli Studi Di Milano, 20100 Milan, Italy; monica.solbiati@unimi.it (M.S.); giorgio.costantino@unimi.it (G.C.); 7 Emergency Medicine Unit, Luigi Sacco Hospital, ASST Fatebenefratelli Sacco, 20100 Milan, Italy; paolo.villa@asst-fbf-sacco.it

**Keywords:** machine learning, syncope, risk stratification, emergency department

## Abstract

Syncope is a challenging problem in the emergency department (ED) as the available risk prediction tools have suboptimal predictive performances. Predictive models based on machine learning (ML) are promising tools whose application in the context of syncope remains underexplored. The aim of the present study was to develop and compare the performance of ML-based models in predicting the risk of clinically significant outcomes in patients presenting to the ED for syncope. We enrolled 266 consecutive patients (age 73, IQR 58–83; 52% males) admitted for syncope at three tertiary centers. We collected demographic and clinical information as well as the occurrence of clinically significant outcomes at a 30-day telephone follow-up. We implemented an XGBoost model based on the best-performing candidate predictors. Subsequently, we integrated the XGboost predictors with knowledge-based rules. The obtained hybrid model outperformed the XGboost model (AUC = 0.81 vs. 0.73, *p* < 0.001) with acceptable calibration. In conclusion, we developed an ML-based model characterized by a commendable capability to predict adverse events within 30 days post-syncope evaluation in the ED. This model relies solely on clinical data routinely collected during a patient’s initial syncope evaluation, thus obviating the need for laboratory tests or syncope experienced clinical judgment.

## 1. Introduction

Increasing evidence suggests that the use of machine learning (ML) algorithms can improve emergency department (ED) triage, diagnosis, and risk stratification for various diseases [1]. However, the lack of external validation and reliable diagnostic standards currently limits their implementation in clinical practice.

Syncope represents a challenging problem for emergency physicians. This is largely due to the fact that its diagnosis frequently lacks the backing of specific tests [2]. Moreover, numerous available prognostic tools [3,4,5,6,7] were found to be partially inefficient [8,9]. In addition, while new and promising risk scores have emerged [10,11,12], their external validation in countries and contexts other than those of derivation showed no significant advantage over ED physicians’ clinical judgment [13,14,15].

Recently, researchers highlighted the potential role of artificial intelligence in managing syncope [16]. However, at present, only a few studies [17,18,19,20] have analyzed the application of ML in syncope risk prediction. Although initial results were promising, these models have yet to undergo external validation to confirm their generalizability and true value in clinical practice.

The aim of the present study is to develop ML-based models to predict 30-day adverse events among patients admitted to the ED for a syncopal episode.

## 2. Methods

### 2.1. Population

The present investigation is a sub-study of The Syncope Monitoring and Natriuretic peptides in the Emergency department (SyMoNE) study [21].

We enrolled 266 patients (age ≥ 18 years), consecutively admitted for syncope to the EDs of three tertiary hospitals in the Milan area (Ospedale Maggiore Policlinico, Luigi Sacco Hospital, Humanitas Research Hospital) from 1 September 2015 to 28 February 2017. The exclusion criteria are detailed elsewhere [21].

For all participants, we recorded demographics, past medical history, vital signs, hemoglobin values, characteristics of the index syncopal episode, and ECG features upon arrival. These were categorized based on the high-/low-risk features established by prior consensus [22] and the ESC guidelines [2]. A detailed list of all collected variables is provided in Table S1 of the Supplementary Materials.

All patients were contacted via telephone for a 30-day follow-up to assess the occurrence of any adverse events. All provided written consent and oral consent to the telephone interviews, as applicable.

### 2.2. Definitions

Syncope is defined as a transient loss of consciousness, likely due to transient global cerebral hypoperfusion, and characterized by a rapid onset, a short duration, and a spontaneous complete recovery [2,23].

Electrocardiogram (ECG) was considered abnormal when presenting new onset non-sinus rhythm or other abnormalities according to previous consensus [22,24].

According to the “Standardized Reporting Guidelines for Emergency Department Syncope Risk-Stratification Research” [24], we considered the following as adverse events at 30 days: death from all causes or related to syncope; ventricular fibrillation; sustained and symptomatic non-sustained ventricular tachycardia; sinus arrest with cardiac pause > 3 s; sick sinus syndrome with alternating bradycardia and tachycardia; second-degree type 2 or third-degree atrioventricular (AV) block; permanent pacemaker (PM) or implantable cardioverter defibrillator (ICD) malfunction with cardiac pauses; aortic stenosis with valve area ≤ 1 cm^2^; hypertrophic cardiomyopathy with outflow tract obstruction; left atrial myxoma or thrombus with outflow tract obstruction; myocardial infarction; pulmonary embolism; aortic dissection; occult hemorrhage or anemia requiring transfusion; syncope or fall resulting in major traumatic injury (requiring admission or procedural/surgical intervention); PM or ICD implantation; cardiopulmonary resuscitation; syncope recurrence with hospital admission; cerebrovascular events.

In this study, we did not consider acute conditions diagnosed in the ED for which syncope was the presenting symptom. However, as the aim of the primary study was to evaluate the diagnostic accuracy of ECG monitoring in non-low-risk patients, adverse events diagnosed during monitoring were included.

### 2.3. Model Development

The raw dataset contains a table with 266 rows and 39 columns. Each row represents the data for a unique patient. Of the 39 columns, one (labeled ‘events’) indicates the outcome, while the remaining columns serve as potential predictors for the models. All predictors and the label are binary in nature. The dataset is imbalanced with respect to the outcome and many features (the predictors) are sparse, i.e., predominantly consisting of zeros. For a detailed breakdown of 0 (false), 1 (true), and missing values, refer to Table S1 in the Supplementary Materials. Three features, specifically “history of congenital heart disease”, “arrhythmogenic right ventricular cardiomyopathy”, and “ECG changes consistent with acute ischemia”, are constants. Since constant features lack predictive value, they were removed from the dataset, leaving 35 candidate predictors. We addressed missing values by substituting them with the most frequent value of the corresponding feature.

**Figure 1:**
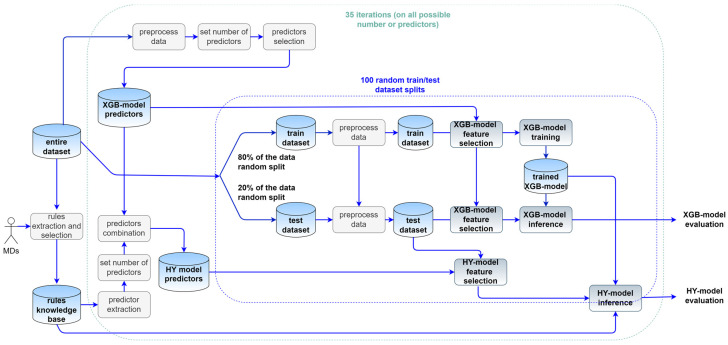
Data pipeline for models development. Abbreviations: MDs, medical doctors; HY model, hybrid model. In the figure cylinders represent data (e.g., Excel or binary files), while rounded rectangles represent blocks of code (e.g., Python scripts).

The data elaboration pipeline is shown in Figure 1.

The entire raw dataset is preprocessed to replace missing data with the most frequent data so that it can be used to identify the best predictors. For each possible number of (model) predictors the best (model) predictors are identified with a chi squared test. The rules knowledge base is obtained through first screening the entire raw dataset to find candidate rules that are subsequently filtered by MDs using clinically plausible criteria. The predictors implicit in the knowledge base rules and the best model predictors are combined to obtain the best hybrid model predictors. The raw data are randomly split into train (80%) and test (20%) data. The train dataset is used to identify the most frequent value and missing values are replaced with the most frequent value in both the train and test dataset (data preprocessing). The best model predictors are used to filter the training and test dataset (feature elimination) for the XGBoost model training and evaluation. Split and evaluation are repeated 100 times. The XGBoost model is used to estimate the event probability and the probability is modified by the hybrid model using the rules knowledge base. Performance of the XGBoost and hybrid model on the test dataset are computed for each possible number of predictors. The highest Matthews Correlation Coefficient (MCC) is used to select the best number and set of XGBoost model and hybrid model predictors.

#### 2.3.1. Rules Knowledge Base

Upon examining the entire raw dataset, we pinpointed all logical rules that adhered to one of the following formats:

IF <antecedent> THEN <increase_predicted_probability>

IF <antecedent> THEN <decrease_predicted_probability>

where the antecedent is the logical conjunctions of any of the 35 candidate predictors. For the sake of interpretability, we limited the number of predictors in the antecedent to a maximum of three. See Table S2 of Supplementary Materials for the rule’s knowledge base in tabular format.

The rules having a potential impact on the model performance were inspected by two physicians who are experts in syncope (FD, RF) with the following objectives:

- Filtering out the rules with unclear clinical interpretability;
- Ranking the predicted probability values as low, medium, and high for both the probability increase and probability decrease;
- Selecting 20 of the rules to ensure the simplicity of the hybrid model.

The rules obtained through this process formed our knowledge base of rules.

#### 2.3.2. Candidate Predictors

The entire (raw) dataset was first analyzed to find the best predictors. For each of the 35 possible number of predictors, we identified the best candidate predictors using a chi squared test. Ideally, this operation would be conducted on a distinct validation dataset, separate from the training and test datasets. However, due to the limited size of our dataset, we utilized the whole dataset for this operation. Out of this operation, we obtained for each of the possible number of predictors a candidate XGBoost model predictors sets. By extracting all predictors used by the rules from the rule knowledge base and incorporating these into the XGBoost model’s predictors, we generated 35 hybrid model predictors sets.

#### 2.3.3. XGBoost Model

We then randomly split the entire (raw) dataset into training and testing datasets (an 80/20% split). In the training dataset, we identified the most common value for each feature. For both training and testing datasets, missing values were replaced with the most common value of the respective feature. The training and testing datasets were subsequently filtered by retaining only the candidate XGBoost model predictors. This filtered training dataset was employed to train and evaluate the XGBoost model.

The block of operations described in the previous paragraph was repeated 100 times with different random/test splits.

**Figure 2:**
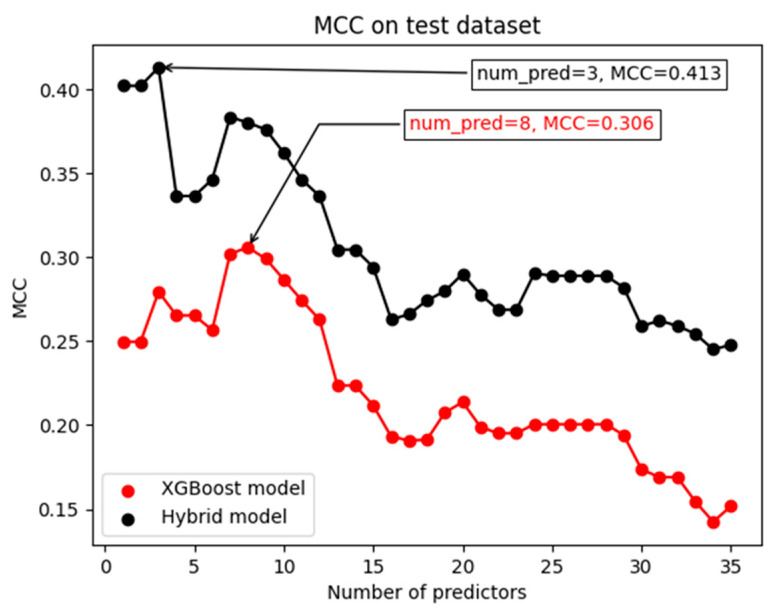
Predictors selection. The figure shows the Matthews Correlation Coefficient (MCC) on the test dataset obtained for each possible number of candidate predictors. The red dots show the performance of the XGBoost models, while the black dots show the performance of the hybrid models. The annotations in the figure show the values at which the curves achieve their maximum value.

The series of steps described in the previous two paragraphs were repeated 35 times, one for each possible candidate XGBoost model predictor set, thereby obtaining the graph in Figure 2 (red points). Using this graph, we determined the optimal set of predictors by choosing the predictor set that achieved the highest Matthews Correlation Coefficient (MCC, see below) on the testing dataset.

XGBoost models were selected because they have top performance on tabular data [25].

#### 2.3.4. Hybrid Model

The hybrid model does not require training; instead, it utilizes previously trained XGBoost models (one for each candidate set of XGBoost model predictors) [26].

We randomly split the entire (raw) dataset into training and testing datasets, employing an 80/20% split procedure. Next, within the training set, we determined the most common value for each feature. Both the training and testing datasets then had any missing values replaced with the respective feature’s most common value. Following that, the training and testing datasets were filtered to retain only the predictors specific to the hybrid model. The filtered training dataset was then used to evaluate the hybrid model.

The procedures outlined in the preceding paragraph were repeated 100 times with different random/test splits.

The sequence of operations described in the two previous paragraphs were conducted 35 times, one for each possible candidate hybrid model predictor set. The results from these iterations are depicted in Figure 2, represented by black points. From this graph we obtained the optimal set of predictors by selecting the set of predictors with the highest MCC on the testing dataset.

#### 2.3.5. XGBoost Model Hyperparameters

XGBoost models performance strongly depends on the selection of a few hyper-parameters. These parameters are often selected by extensive search on the validation dataset. Due to the smallness of the dataset, we could not afford having a validation dataset and therefore we decided to perform search on the entire dataset. The most important parameters are described hereafter.

The parameter ‘scal_pos_weight’ controls the balance of positive and negative weights. Since our dataset is imbalanced (event = True much rarer than event = False), we had to increase it from the default value (1) to the 5.2 value. The parameter ‘n_estimators’ controls the number of boosted trees to fit. The default value is 100 but seeing that our dataset is very small we used the much lower value of 7. The parameter ‘max_depth’ controls the maximum depth of a tree. The default value is 7, but to reduce overfitting we used the value of 6.

### 2.4. Data Analysis

Descriptive data are presented as median (with interquartile range—IQR) for continuous variables, and as numbers and percentages for categorical variables for describing baseline characteristics of all enrolled patients and 30-day adverse events.

To evaluate the model predictive performance, we assessed discrimination and calibration.

As measures of discrimination, we calculated F1 score, area under the curve (AUC), and Matthews Correlation Coefficient (MCC), which has intrinsic ability to simultaneously consider true positive, false positive, true negative, and false negative predictions, making it more reliable for binary classification tasks [27,28]. MCC ranges from −1 to +1, with 0 representing a prediction no better than random. The F1 score, AUC, and MCC of different models have been compared using a paired *t*-test.

Model calibration was assessed using the expected calibration error (ECE) [29]. Calibration discretizes the probability interval into a fixed number of bins and assigns each predicted probability to the bin that encompasses it. The ECE is the difference between the fraction of correct predictions in the bin (accuracy) and the mean of the probabilities in the bin (confidence). Therefore, if a model’s accuracy is equal to the bin’s mean probability, the ECE would be 0, indicating perfect calibration. Lower values of ECE correspond to better calibrations.

## 3. Results

**Figure 3:**
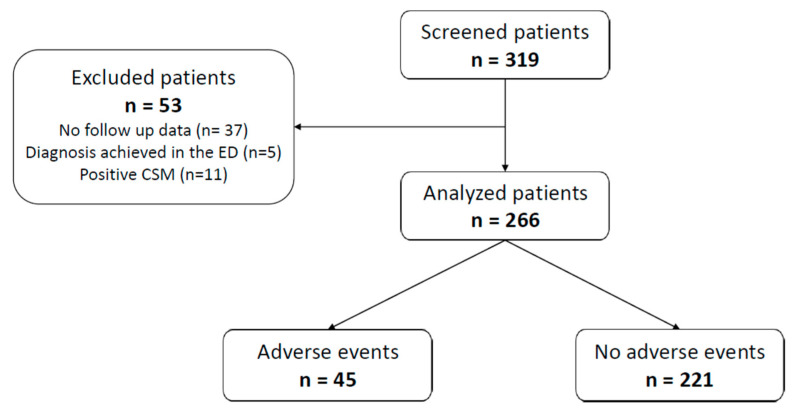
Study flow chart. Abbreviations: CSM, carotid sinus massage.

During the enrollment period, the researchers screened 319 patients who presented to the three participating hospitals’ EDs for syncope for potential study participation. A total of 266 patients were included in the study (Figure 3).

Table 1 shows the demographic and clinical characteristics of the study population.

After 30 days of follow-up, 45 (16.9%) patients had serious outcomes and three of these patients died (see Table 2).

Table 3 summarizes the predictors included in the XGBoost model and the hybrid model.

Table 4 summarizes the performance of XGBoost and hybrid models in predicting syncope 30-day adverse events in the test set.

The hybrid model showed significantly greater discrimination capability than the XG boost model (*p* < 0.001).

**Figure 4:**
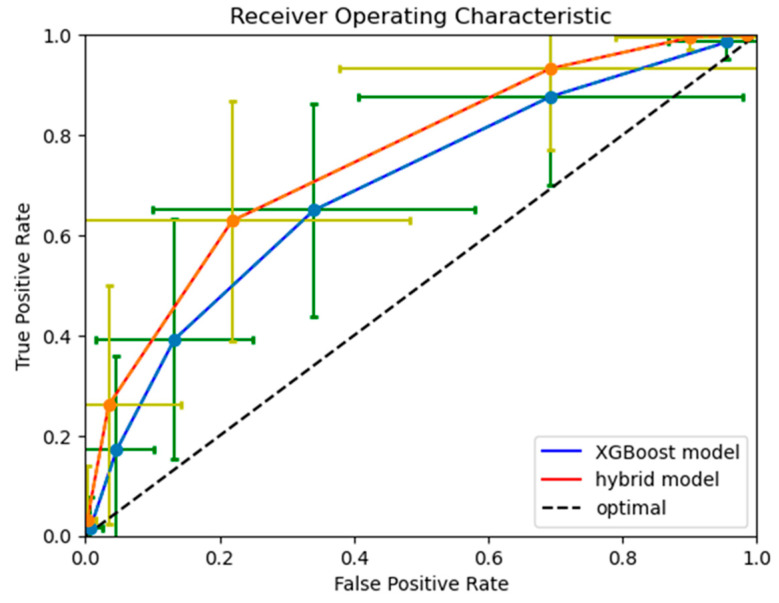
XGBoost model and hybrid model receiver operating characteristic curves. The blue and orange curves show the ROC curve for the XGBoost and hybrid model, respectively. Both curves show the values obtained through taking the average on the 100 iterations. The green and yellow vertical bars show the standard deviation (on the same 100 iterations) of the true positive rate; while the green and yellow horizontal bars show the standard deviation of the false positive rate.

Both models’ receiver operating characteristic (ROC) curves are displayed in Figure 4.

The mean ECE for outcome prediction was 0.442 ± 0.032 for the XGBoost model and 0.483 ± 0.034 for the hybrid model.

## 4. Discussion

In this study, we determined that several factors, including age under 40 years, history of heart failure, prior instances of ischemic heart disease, previous pulmonary hypertension, being equipped with an ICD, recurrence of syncope within the last year, a heart rate below 40 bpm, and the presence of either a second-degree heart block Mobitz type II or third-degree heart block on the ECG, were effective in predicting the likelihood of adverse events at 30 days for patients assessed in the ED for syncope. These predictors achieved an AUC of 0.73 and an MCC of 0.32. After accounting for combined predictors through clinical rules, the model’s performance enhanced, as indicated by an AUC of 0.80 and an MCC of 0.43.

The hybrid model in question integrates an ML model (specifically, XGBoost) with a series of logical rules. When applied, these rules have the potential to modify the resulting predictions and associated probabilities. These logical rules were ascertained by meticulously analyzing the complete dataset to find relevant rules. Subsequently, we excluded rules that either lacked clinical clarity or had a minimal effect on the model’s efficacy. Each retained rule was then ranked based on its perceived clinical significance, with its importance being graded as low, medium, or high, contingent on the projected probability of an adverse event as determined by the researchers’ clinical expertise.

Rules that increase the likelihood of experiencing adverse events from syncope within 30 days incorporate the following risk factors: age above 40 years; syncope during exertion; syncope in seated position; syncope not in orthostatic position; absence of syncopal recurrences in the last year; history of ischemic cardiomyopathy; history of congestive heart failure; history of left ventricle (LV) ejection fraction below 40%; history of pulmonary hypertension; history of arterial hypertension; heart rate below 40 bpm; ECG abnormal.

Rules that decrease the likelihood of syncope 30-day adverse events include the following protective factors: syncope triggered by pain/stressors; syncope triggered by cough, micturition, defecation; syncope while standing from a seated position; syncope associated with nausea/vomiting; syncope associated with sensation of warmth; presence of syncopal recurrences in the last year; no history of congestive heart failure, arterial hypertension, LV ejection fraction below 40%; no family history of sudden death; SBP > 90 mm Hg; ECG normal at presentation; absence of (new) non sinus rhythm.

These findings indicate that by solely utilizing data related to the presentation of the syncope episode, a patient’s medical history, vital signs, and ECG—all of which a clinician can easily gather during the initial assessment of a patient with syncope—it is feasible to predict 30-day adverse events with good discrimination and reliable calibration.

Recently, a study by Grant et al. [17] presented a gradient-boosting (GB) model adept at forecasting 30-day adverse events for syncope patients after ED disposition. Drawing from all 43 variables considered during the derivation phase of the Canadian Syncope Risk Score [11], they pinpointed several predictors: age; ED diagnosis of cardiac syncope; ED diagnosis of vasovagal syncope; history of heart disease; QRS duration; QRS axis; QTc > 480 ms; troponin levels above 99% of the normal population; and hemoglobin levels. This GB model achieved predictions for a 30-day composite endpoint similar to ours, encompassing most of the adverse events we assessed, and boasted an AUC of 0.91 with acceptable calibration. While our model exhibits lower performance and requires a larger set of predictors, it holds a distinct advantage: it can be seamlessly implemented in diverse clinical environments—not just the ED, given its exclusion of lab tests. Moreover, our model hinges on predictors linked to the patient’s history, physical examination, and ECG readings at the outset, and is not influenced by the subjective expertise of a physician’s diagnosis of cardiac or vasovagal syncope. Notably, the term “cardiac syncope” itself encompasses various conditions that align with the exact outcomes our model seeks to forecast. Conversely, vasovagal syncope, by its very nature, is deemed a benign condition that does not warrant risk prediction. As such, incorporating a syncope’s etiological diagnosis, even if solely based on an emergency physician’s initial assessment, might diminish the predictive model’s clinical relevance and escalate the overfitting risk [30].

While we acknowledge a certain degree of subjectivity and plausible inaccuracy in the anamnestic collection [31] from the patient with syncope, especially if amnesic about the event and in the absence of witnesses, we believe that most of the anamnestic information used as predictors in our model is objectifiable and easily obtained by even the least-experienced physician.

In an earlier study, Costantino et al. [19] developed an artificial neural network (ANN) to predict the short-term prognosis of syncope. The predictors they utilized included sex, age, syncope during exertion, trauma following syncope, presence of abnormal ECG, absence of prodromes, history of cerebrovascular disease, history of cardiac disease, and history of hypertension. This ANN, when tested on a cohort of 1844 patients from three independent prospective studies [4,6,32], proved to predict a previously established composite endpoint [9,23] 7–10 days after -ED evaluation. The AUC varied between 0.69 and 0.78, depending on the proportion of patients used in the training and test sets.

While there are significant similarities in terms of the predictors used, the clinical setting, and the serious outcomes predicted, and even with comparable performance metrics, we do believe that our approach holds an advantage. Specifically, we opted not to include acute conditions diagnosed in the ED within the composite endpoint, as we view such predictions as having limited clinical relevance. Indeed, when the cause of syncope is evident after initial assessment, the subsequent steps and potential treatment strategies in the ED are well-established by available guidelines [2]. However, whenever the cause of syncope remains elusive, a precise prognostic stratification becomes crucial. It ensures that low-risk patients, who are unlikely to experience adverse events within 30 days, can be safely discharged, while high-risk patients, who might require extended monitoring or immediate treatments, have not to be prematurely released.

The same authors [20] also demonstrated that such ANN could predict patient hospitalization with an AUC between 0.79 and 0.89, thus outperforming previous predictive tools based on traditional statistical methods, in turn resulting in possible increased appropriateness of care and enhanced hospital efficiency.

Using a large US administrative database that encompassed nearly 5 million patients across 37 states, and included demographics as well as data on 31 comorbidities, Lee et al. [18] recently devised an ANN capable of predicting the length of hospital stays. The AUC for this prediction ranged between 0.78 and 0.88, varying based on the specific time thresholds implemented. While the length of stay might be considered as an indicator of disease severity, it is worth noting that factors such as the rationale for hospital admission and duration of hospitalization can also be influenced by socioeconomic considerations and structural elements inherent to different healthcare systems. The authors themselves acknowledged the intrinsic drawbacks of retrospectively analyzing an administrative database, namely the absence of clinically relevant data such as the results from diagnostic tests.

In the past few years, ANNs, along with their extension, deep learning (DL) models, have been rigorously and successfully evaluated in diverse clinical settings. Their strength lies in processing vast amounts of data and recognizing nonlinear correlations between risk factors (inputs) and the outcomes they are designed to predict (outputs), thereby mimicking the functioning of the human nervous system [33,34]. However, it is essential to highlight that the lack of explainability, interpretability, and traceability may lead clinicians to distrust these “black box” models and prefer linear “white box” models that can clearly demonstrate how they produce predictions and which input features are influential (e.g., linear regression, gradient-boosting decision trees) [16].

## 5. Study Limitations

We acknowledge that our study presents certain limitations. Firstly, the dataset we employed is relatively small with a notably low event-per-variable ratio [35,36]. As a result, there is a potential that our model’s apparent performance metrics might be biased. However, calculating the average value of the MCC over 100 iterations, in relation to the increasing percentage of data used, we observed that the MCC remained largely stable, while its standard deviation (SD) decreased more notably (see Figure S1 in Supplementary Materials). Therefore, we inferred that using a larger sample size would not have significantly altered the predictive performance of our model, but it could potentially lead to a more stable estimation of the results. Currently, data limitations stand as a significant barrier to the advancement of ML-based predictive tools. However, it is likely that in the future these limitations may be overcome by the formation of large, shared, prospective clinical databases. Additionally, like other ML-based predictive models cited above, our model has only been evaluated within its development cohort. We are aware that external validation is necessary to assess its generalizability. Another potential limitation of our study lies in our subjective weighting of the various clinical rules incorporated into our hybrid model, which may not be universally shared among researchers. Lastly, our choice to integrate our model’s predictors with clinical rules, i.e., combinations of risk or protective factors, may limit usability and portability. However, given that the required information is part of the standard initial assessment of syncope and is obtainable in any clinical setting, and that currently user-friendly calculators and information technology (IT) solutions are within everyone’s reach, we do believe that this is not a real impediment to its possible future implementation in daily clinical practice.

## 6. Conclusions

In the present study, we aimed to explore the potential of supervised ML-based models in automating the risk stratification process of the patient with syncope.

We developed a hybrid model characterized by a commendable capability to predict adverse events occurring within 30 days post-syncope evaluation in the ED. This model relies solely on patient history, vital signs, and the ECG at presentation, obviating the need for laboratory tests or syncope experienced clinical judgment. While encouraging, our findings are far from being conclusive.

In the foreseeable future, ML-based predictive models might offer a promising alternative to traditional syncope risk stratification methodologies, which have shown in the past a limited effectiveness. To advance and operationalize dependable and clinically pertinent predictive models, it is crucial to establish expansive, collaborative prospective clinical databases, prioritize high-quality data acquisition, and foster cooperation between clinicians, data scientists, and IT specialists. Moreover, given that a bulk of intricate prognostic data exists primarily in textual format, a significant challenge will involve leveraging natural language processing on electronic health records to extract pertinent phenotypic data. Finally, the implementation of unsupervised ML methods could potentially uncover currently unknown risk patterns, leading, as a result, to more personalized approaches in risk prediction.

## Figures and Tables

**Table 1:** Characteristics of enrolled patients.

Characteristic	*n* (%) or Median (IQR)
Patients enrolled	266
Patient characteristics	
Sex (male)	139 (52.3)
Age (years)	73 (58–83)
Systolic blood pressure (mm Hg)	130 (110–150)
Systolic blood pressure < 90 mm Hg	7 (2.6)
Heart rate (beats/min)	75 (65–86)
Admitted to hospital	90 (33.8)
Syncopal episode characteristics	
During exertion	3 (1.1)
In supine position	8 (3)
In seated position	74 (27.8)
In orthostatic position	116 (62.4)
While standing from a seated position	13 (4.9)
Associated with	
Chest pain	14 (5.3)
Palpitations	13 (4.9)
Nausea/vomiting	52 (19.5)
Sensation of warmth	24 (9)
Triggered by pain/stressors	14 (5.3)
Triggered by cough/micturition/defecation	18 (6.8)
Past medical history	
Syncope in the previous year	71 (26.7)
Family history of sudden death	7 (2.6)
Congestive heart failure	9 (3.4)
Ischemic cardiomyopathy	46 (17.3)
Congenital heart disease	0 (0)
Aortic stenosis	5 (1.9)
Left ventricular outflow obstruction	1 (0.38)
Dilated/hypertrophic cardiomyopathy	4 (1.5)
Left ventricular ejection fraction < 40%	6 (2.3)
Pulmonary hypertension	9 (3.4)
Previously documented arrhythmia (ventricular)	2 (0.75)
Previous ICD implantation	2 (0.75)
Arterial hypertension	151 (56.8)
Stroke/TIA	24 (9)
Neoplasm	37 (13.9)
Chronic kidney disease (serum creatinine ≥ 2 mg/dL)	12 (4.5)
COPD	16 (6)
ECG findings (ECG results available for 258 patients)	
Normal	224 (86.8)
Non-sinus rhythm (new)	12 (4.6)
New (or previously unknown) left bundle branch block	8 (3.1)
Bifascicular block	2 (0.78)
Bifascicular block + first degree AV block	8 (3.1)
High-grade (second-degree type 2 or third-degree) AV block	3 (1.2)
Sinus bradycardia (≤50 bpm)	11 (4.3)
Prolonged QTc (>450 ms)	5 (1.9)
Brugada ECG pattern	1 (0.39)
Arrhythmogenic right ventricular cardiomyopathy	0 (0)
ECG changes consistent with acute ischemia	0 (0)

Abbreviations: ICD, implantable cardioverter–defibrillator; TIA, transient ischemic attack; COPD, chronic obstructive pulmonary disease; AV block, atrioventricular block; ECG, electrocardiogram.

**Table 2:** Thirty-day adverse events.

Adverse Event	*n* (%)
Serious adverse events	45 (16.9)
Death	3 (1.1)
Ventricular fibrillation	1 (0.04)
Cardiac pause > 3 s/third-degree AV block	3 (1.1)
PM/ICD malfunction with cardiac pauses	1 (0.04)
PM or ICD implantation	22 (8.3)
Syncope recurrence with hospital admission	7 (2.6)
Myocardial infarction	1 (0.04)
Pulmonary embolism	1 (0.04)
Occult hemorrhage or anemia requiring transfusion	4 (1.5)
Cerebrovascular event	2 (0.08)

Abbreviations: AV block, atrioventricular block; PM, pacemaker; ICD, implantable cardioverter–defibrillator.

**Table 3:** Models predictors.

XGBoost Model	Hybrid Model
Age < 40 years Syncopal recurrences in the last year History of ischemic cardiomyopathy History of congestive heart failure History of pulmonary hypertension History of previous ICD implantation Second-degree type 2 or third-degree AV block Heart rate < 40 bpm	XGBoost model predictors Syncopal recurrences in the last year History of previous ICD implantation Heart rate < 40 bpm *combined with* Knowledge base predictors Age < 40 years Syncope during exertion Syncope in seated position Syncope while standing from a seated position Syncope in orthostatic position Syncope associated with nausea/vomiting Syncope associated with sensation of warmth Syncope triggered by pain/stressors Syncope triggered by cough/micturition/defecation Syncopal recurrences in the last year History of ischemic cardiomyopathy History of congestive heart failure History of LV ejection fraction < 40% History of pulmonary hypertension History of arterial hypertension Family history of sudden death Systolic blood pressure < 90 mm Hg Heart rate < 40 bpm ECG normal Non sinus rhythm (new)

Abbreviations: AV block, atrioventricular block; LV, left ventricle.

**Table 4:** Model performances in predicting 30-day adverse events from syncope ED evaluation.

Model	F1 Score	AUC	MCC
XG Boost	0.637 ± 0.053	0.728 ± 0.073	0.318 ± 0.110
Hybrid	0.701 ± 0.056 *	0.801 ± 0.060 *	0.430 ± 0.114 *

Results are presented as means and standard deviations of 100 iterations. AUC, area under the curve; MCC, Matthews Correlation Coefficient. * *p* < 0.001 according to a paired *t*-test to compare each performance metric between the two groups.

## Data Availability

Data are contained within the article and Supplementary Materials.

## Supplementary Materials

The following supporting information can be downloaded at: https://www.mdpi.com/article/10.3390/jpm14010004/s1, Table S1. Candidate predictors, data sparsity and missing. Table S2. Rules knowledge base. Figure S1. Models’ performances on test dataset as a function of the sample size.
