# Supplementary material for: A Hybrid Model for 30-Day Syncope Prognosis Prediction in the Emergency Department
Source: J Pers Med. 2023 Dec 20;14(1):4. doi: 10.3390/jpm14010004 (PMC10817569; doi:10.3390/jpm14010004)
Supplement: Supplementary file 1 [file jpm-14-00004-s001.zip › jpm-2709905-supplementary.pdf]

Table S1. Candidate predictors, data sparsity and missing

	Values = 0	Values = 1	Values = missing
Age < 40 years	244	22	0
Systolic blood pressure < 90 mmHg	258	7	1
Heart rate < 40 bpm	263	2	1
Hemoglobin < 9 g/dl	253	10	3
Syncope during exertion	263	3	0
Syncope in supine position	258	8	0
Syncope in seated position	192	74	0
Syncope in orthostatic position	100	166	0
Syncope while standing from a seated position	253	13	0
Syncope associated with chest pain	252	14	0
Syncope associated with palpitations	253	13	0
Syncope associated with nausea/vomiting	214	52	0
Syncope associated with sensation of warmth	242	24	0
Syncope triggered by pain/stressors	252	14	0
Syncope triggered by cough/micturition/defecation	248	18	0
Family history of sudden death	259	7	0
Syncope in the previous year	195	71	0
History of arterial hypertension	115	151	0
History of congestive heart failure	257	9	0
History of ischemic cardiomyopathy	220	46	0
History of congenital heart disease	266	0	0
History of aortic stenosis	261	5	0
History of left ventricular outflow obstruction	265	1	0
History of dilated/ hypertrophic cardiomyopathy	262	4	0
History of left ventricular ejection fraction < 40%	260	6	0
History of pulmonary hypertension	257	9	0

History of previously documented arrhythmia (ventricular)	264	2	0
History of previous ICD implantation	264	2	0
ECG normal	34	224	8
Non-sinus rhythm (new)	246	12	8
New (or previously unknown) left bundle branch block	250	8	8
Bifascicular block	256	2	8
Bifascicular block + first degree AV block	250	8	8
High-grade (second-degree type 2 or third-degree AV block	255	3	8
Prolonged QTc (>450 ms)	253	5	8
Brugada ECG pattern	257	1	8
Arrhythmogenic right ventricular cardiomyopathy	258	0	8
ECG changes consistent with acute ischemia	258	0	8
Events	221	45	0

Abbreviations: ICD, implantable cardioverter defibrillator; ECG, electrocardiogram; AV block, atrioventricular block; QTc, corrected QT interval

Table S2. Rules knowledge base

Predictor #1	Predictor #2	Predictor #3	Coefficient
**Rules that increase the likelihood of syncope 30-day adverse events**			
**Predictor #1**	**Predictor #2**	**Predictor #3**	**Coefficient**
Syncope during exertion = 1	Age < 40 years = 0	-	0.2
Syncope during exertion = 1	History of ischemic cardiomyopathy = 1	-	0.3
Syncope in seated position = 1	History of ischemic cardiomyopathy = 1	History of congestive heart failure = 1	0.3
Syncope in seated position = 1	History of congestive heart failure = 1	History of pulmonary hypertension = 1	0.3
Syncope in seated position = 1	History of congestive heart failure = 1	Syncope in the previous year = 0	0.2
Syncope in orthostatic position = 0	History of ischemic cardiomyopathy = 1	ECG normal = 0	0.3
Syncope in orthostatic position = 0	History of ischemic cardiomyopathy = 1	History of pulmonary hypertension = 1	0.3
History of congestive heart failure = 1	History of LV ejection fraction < 40% = 1	History of arterial hypertension = 1	0.3
Heart rate < 40 bpm	-	-	0.1
**Rules that decrease the likelihood of syncope 30-day adverse events**			
Syncope triggered by pain/stressors = 1	Syncope associated with nausea/vomiting = 1	-	0.2
Syncope triggered by pain/stressors = 1	Syncope in the previous year = 1	-	0.2
Syncope triggered by pain/stressors = 1	Family history of sudden death = 0	-	0.1
Syncope triggered by pain/stressors = 1	Syncope while standing from a seated position = 1	-	0.3
Syncope while standing from a seated position = 1	ECG normal = 1	-	0.3
Syncope while standing from a seated position = 1	Non-sinus rhythm (new) = 0	-	0.1
Syncope while standing from a seated position = 1	Syncope associated with nausea/vomiting = 1	-	0.3
Syncope triggered by cough/micturition/defecation = 1	History of congestive heart failure = 0	-	0.1

Syncope triggered by cough/micturition/defecation = 1	Syncope in the previous year = 1	Systolic blood pressure < 90 mmHg = 0	0.2
Syncope triggered by cough/micturition/defecation = 1	Syncope in the previous year = 1	History of LV ejection fraction < 40% = 0	0.3
Syncope associated with sensation of warmth = 1	Family history of sudden death = 0	History of arterial hypertension = 0	0.1

Abbreviations: ECG, electrocardiogram

Figure S1. Models performance on test dataset as a function of the sample size

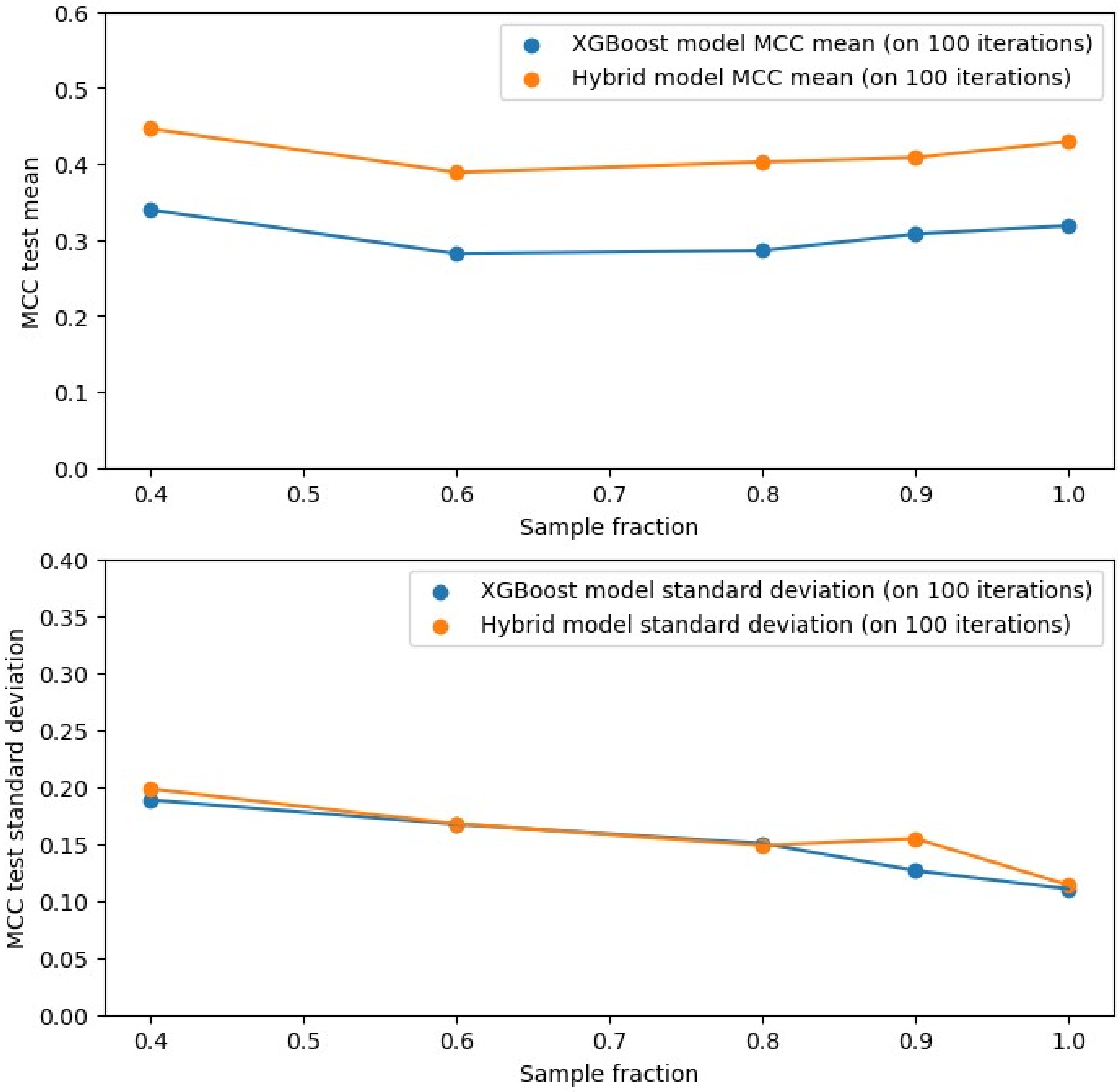

The figures are obtained by randomly sampling the rows of the entire dataset. Each value in the abscissa axis corresponds to a percentage of data (e.g., 0.40 means we took 40% of the data) and therefore to a specific sample. This sample is used to compute the average of the Matthews Correlation Coefficient (MCC) on 100 iterations. This value is plotted on the top figure, while the bottom figure shows the corresponding standard deviation (SD). Each plot shows the values obtained with the XGBoost and hybrid models.
